# The normal environment delays the development of multisensory integration

**DOI:** 10.1038/s41598-017-05118-1

**Published:** 2017-07-06

**Authors:** Jinghong Xu, Liping Yu, Benjamin A. Rowland, Barry E. Stein

**Affiliations:** 1 Key Laboratory of Brain Functional Genomics (Ministry of Education and Shanghai), School of Life Science, East China Normal University, Shanghai, 200062 China; 2 Department of Neurobiology and Anatomy, Wake Forest School of Medicine, Winston-Salem, NC 27157 USA

## Abstract

Multisensory neurons in animals whose cross-modal experiences are compromised during early life fail to develop the ability to integrate information across those senses. Consequently, they lack the ability to increase the physiological salience of the events that provide the convergent cross-modal inputs. The present study demonstrates that superior colliculus (SC) neurons in animals whose visual-auditory experience is compromised early in life by noise-rearing can develop visual-auditory multisensory integration capabilities rapidly when periodically exposed to a single set of visual-auditory stimuli in a controlled laboratory paradigm. However, they remain compromised if their experiences are limited to a normal housing environment. These observations seem counterintuitive given that multisensory integrative capabilities ordinarily develop during early life in normal environments, in which a wide variety of sensory stimuli facilitate the functional organization of complex neural circuits at multiple levels of the neuraxis. However, the very richness and inherent variability of sensory stimuli in normal environments will lead to a less regular coupling of any given set of cross-modal cues than does the otherwise “impoverished” laboratory exposure paradigm. That this poses no significant problem for the neonate, but does for the adult, indicates a maturational shift in the requirements for the development of multisensory integration capabilities.

## Introduction

At least two interrelated factors appear to be necessary for superior colliculus (SC) neurons to develop their multisensory integration capabilities: functional inputs from ipsilateral association cortex, and experience with cross-modal events^1^. Thus, deactivating the tectopetal inputs from association cortex during early life disrupts this maturational process^2, 3^, as does precluding relevant (e.g., visual-auditory) experience by rearing the animals in darkness or with continuous masking noise^4–7^. In each of these cases SC neurons fail to show their characteristic enhanced multisensory responses: their responses to a visual-auditory stimulus combination are no greater than those to one of the component stimuli alone.

However, multisensory integration capabilities could be acquired in each of these cases later in life if animals obtained appropriate cross-modal experience. For example, animals reared in darkness developed multisensory integration capabilities within a few weeks of exposure to spatiotemporally concordant flashing lights and noise bursts during weekly sessions in the laboratory^5^. The animals were housed in darkness at all other times. In contrast, when tectopetal inputs from association cortex were pharmacologically deactivated for several weeks during early life, SC neurons later acquired multisensory integration capabilities only after years of experience in a normal animal housing facility^2^. It is curious that experience in the normal housing condition, which is rich in a variety of visual-auditory events, was far less effective in instantiating multisensory integration capabilities than were the weekly exposure sessions. Yet, the latter provided only a single set of cross-modal stimuli, the animals were anesthetized during each exposure period, and they were housed in an otherwise “impoverished” sensory environment.

There are two likely explanations for these different findings, one based on the restriction methods and the other on the “learning” environment. Pharmacological deactivation may have been so disruptive to the activity-dependent maturation of association cortex that it required years of rehabilitation before that cortex became capable of playing its normal role in this SC process. Alternatively, the key difference may reflect a maturational change in the brain’s ability to deal with statistical irregularities in cross-modal experience. Cross-modal cues derived from the same event in normal environments vary in their exact spatiotemporal relationships on different occurrences, can appear under very different background conditions, and are often interleaved with their unpaired counterparts. This variability is avoided in the laboratory exposure paradigm. The present study examined these alternatives by using the same adult animals to compare the effects of the laboratory exposure paradigm and normal environments on the acquisition of SC multisensory integration capabilities.

## Results

A total of 295 overtly responsive visual-auditory multisensory neurons were evaluated. For comparative purposes, additional samples (n = 97) from a dataset previously acquired from normally-reared animals^6^ were included. These neurons had been tested using procedures identical to those used to acquire the present data set.

**Figure 1:**
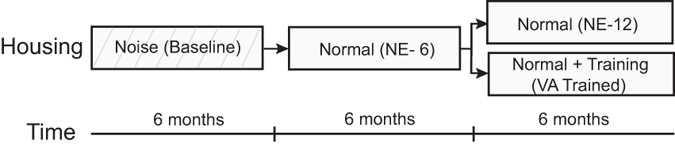
Experimental design. The Experimental timeline shows the periods in which noise-rearing (noise), normal housing (normal), and visual-auditory cross-modal exposure (VA Trained) took place.

Figure 1 illustrates the experimental design. All animals were reared from birth to 6 months of age in an omnidirectional noise room. At this time 32 neurons were examined and their evaluation verified the effectiveness of the noise-rearing condition in blocking the development of multisensory integration capabilities as previously described^6^. Their responses also established a multisensory baseline.

**Figure 2:**
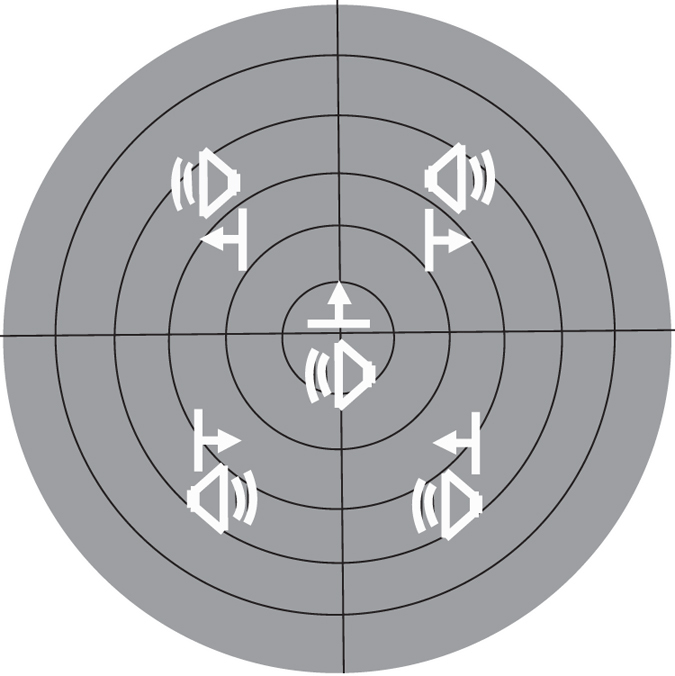
Cross-modal exposure sites. During exposure sessions, animals in the VA Trained group were presented with spatiotemporally concordant visual-auditory stimuli that appeared randomly at one of 5 locations as shown by the two icons (a speaker and a bar of light moved in the direction of the arrow) on a schematic of the central 60° of space.

Animals were then transferred to the normal housing condition for an additional 6 months, after which 68 neurons were examined to determine the effect of this 6 month period of normal experience (NE-6), and thereby determine whether this experience had resolved the multisensory defects induced by noise-rearing. It had not, and animals were then divided into two groups and tested weekly for the next 6 months. Both groups lived in the normal environment, but as noted in Methods, one group, the VA Trained group (n = 3), had weekly exposure to spatiotemporally concordant cross-modal cues after being anesthetized and before each recording session (Fig. 2), the other (NE-12, n = 2) did not. The RF properties and response characteristics of SC neurons in these animals were evaluated in two ways: as they were being acquired on a weekly basis, and in summary form by collapsing all the data obtained in each group during the last 1.5 months of this final phase.

**Figure 3:**
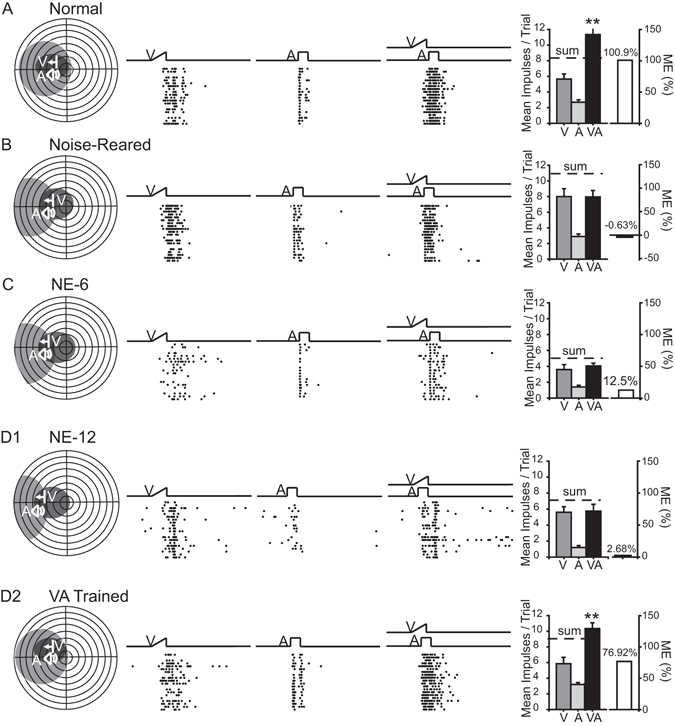
Exemplar neurons illustrating the results of cross-modal testing in various conditions. (**A**) The normal profile of multisensory enhancement in superior colliculus (SC) neurons. Left: a neuron’s visual (dark gray) and auditory (light gray) receptive fields (RFs), and the positions of the visual and auditory stimuli, are shown on the schematic. Conventions are the same as in Fig. 2. Middle: rasters (trials ordered bottom-to-top), show responses to visual (V), auditory (**A**) and spatiotemporally concordant visual-auditory (VA) stimuli. Electronic traces of the stimuli are shown above the rasters. Right: A summary graph of the mean response magnitudes and the multisensory enhancement index (ME) show that the multisensory response significantly exceeded the strongest unisensory comparator response (V) in this normal exemplar. (**B**) With omnidirectional noise-rearing, however, RF register was less precise, and the VA response was no greater than the V response in this baseline condition. (**C**) After 6 months of subsequent experience in a normal housing environment (NE-6), the typical multisensory SC neuron showed little change from baseline. RF overlap was still less precise than normal and no capacity for multisensory enhancement was evident. Its response to the cross-modal stimulus was no greater than to one of the component stimuli. (**D1**) This profile was little changed after an additional 6 months of housing in the normal environment (NE-12). (**D2**) In contrast, after weekly visual-auditory cross-modal exposure sessions conducted during the same period, the typical neuron exhibited good RF register as well as multisensory enhancement capabilities (VA Trained). Sum = the addition of the two unisensory responses. Error bars indicate SEM. **P ≤ 0.001.

The main findings of the experimental series are summarized by the exemplars provided in Fig. 3. In normally-reared control animals (n = 97), visual and auditory RFs contract into register and neurons exhibit characteristic multisensory integration capabilities (Fig. 3A). In contrast, neurons in noise-reared animals (i.e., baseline, n = 32) had much poorer visual-auditory RF register and did not exhibit multisensory integration capabilities (Fig. 3B). These findings are in consistent with previously observations that noise-rearing disrupts the development of SC visual-auditory multisensory integration^6^.

The defects in RF register and multisensory enhancement were not resolved in SC neurons (n = 68) after providing animals with 6 months of normal experience (NE-6) (Fig. 3C), nor were they resolved in SC neurons (n = 23) by extending this experience by an additional 6 months (NE-12) (Fig. 3D1). However, both defects were resolved within this time frame in neurons (n = 34) in the VA Trained group; that is, when cross-modal exposures were explicitly provided prior to each recording session (Fig. 3D2). Each of these differences in RF properties and multisensory enhancement capabilities, observed at each experimental stage and in each condition, is detailed quantitatively below.

### Receptive Field Properties

**Figure 4:**
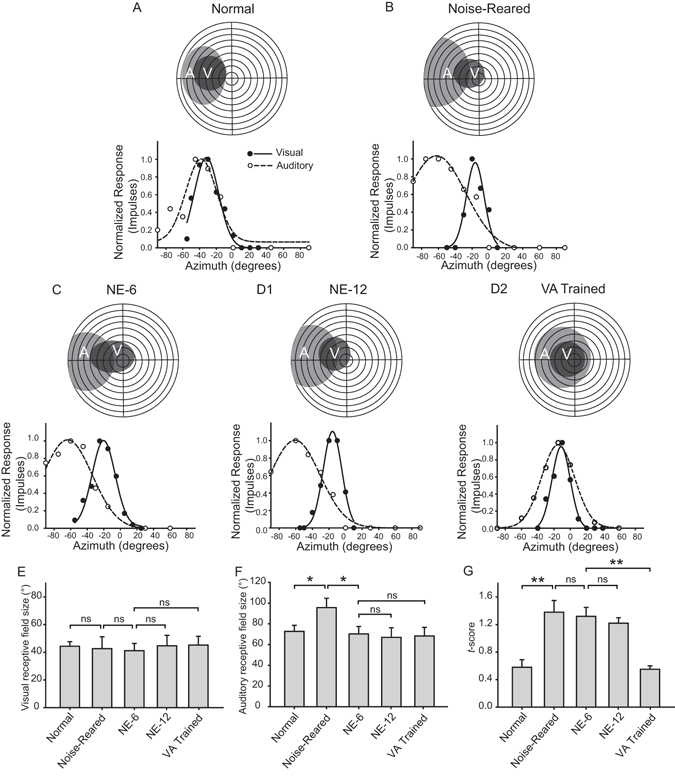
Visual-auditory receptive field register increases with cross-modal exposure. (**A**) The classically-defined visual and auditory RFs of a representative neuron from a normally reared animal are shown at the top, with the visual (solid line) and auditory (dashed line) spatial response profiles shown below (y-axis = mean # impulses/response, normalized by maximum). Note the good register between the RFs using either metric. (**B**) However, this cross-modal RF register was very poor in most neurons of noise-reared animals during baseline tests, and is illustrated by the exemplar. (**C**) Poor cross-modal RF register remained evident even after 6 months of subsequent housing in the normal environment (NE-6). (**D1**) The poor RF registers remained even after an additional 6 months in the normal housing condition (NE-12). (**D2**) However, animals given weekly cross-modal exposure sessions (VA trained) over the same period showed a significant improvement in RF register. (**E**, **F** and **G**) Bar graphs summarize the average size (diameter) of the visual and auditory RFs in the population, and the *t*-scores (higher = greater RF misalignment, see text) in the five conditions. Error bars indicate SEM, ns = non-significant. Other conventions are the same as in previous figures.

The differences in the size and overlap of the RFs of neurons in the different conditions, evident in the exemplars in Fig. 3, were paralleled by differences in their spatial response profiles (i.e., sensitivity to visual and auditory stimuli at different positions along the azimuth). These spatial response profiles provided greater quantitative insight into the internal structure of the multisensory RFs, and their differences among the groups are illustrated by representative exemplars in Fig. 4A–D. Whereas visual and auditory RFs and their spatial response profiles showed good register in normal animals (Fig. 4A), their disruption in noise-reared animals was evident from the baseline tests shown in Fig. 4B. The lack of good register in the spatial profiles of these neurons persisted even after the noise-reared animals were transferred to a normal environment for 6 months (NE-6, Fig. 4C), and remained evident even after an additional 6 months in that environment (NE-12, Fig. 4D1). However, the spatial profiles show that RF register improved significantly after controlled cross-modal exposure during the same period (VA Trained, Fig. 4D2). These observations reflected the population trends as discussed below.

#### Receptive Field Size

Noise-rearing appeared to have little effect on the maturational contraction of visual RFs. These RFs achieved their normal size by the time the baseline data were acquired (mean diameter = 42.66 ± 28.55° in noise-reared versus 44.36 ± 10.51° in normal animals, Mann-Whitney U tests, P = 0.335, Fig. 4E). In contrast, auditory RFs were significantly larger than normal at the time the baseline data were acquired (mean diameter = 95.67 ± 28.98° in noise-reared animals vs. 72.75 ± 25.87° in normal animals, Mann-Whitney U tests, P = 0.002, Fig. 4F). A more detailed analysis revealed that this difference was strongly influenced by a minority (25%, 8/32) of noise-reared neurons that retained the omnidirectional RFs characteristic of the neonate. This type of RF was uncommon in normally reared animals (9% of the normally-reared population). In the remaining noise-reared neurons (75%, 24/32), baseline auditory RFs had contracted to approximately normal size.

As expected, subsequent housing in a normal environment for 6 months had little effect on the visual RFs, as they were already of normal size when the baseline data were collected (mean diameter = 41.17 ± 21.24° at NE-6), but it eliminated the abnormally high incidence of omnidirectional RFs in the noise-reared population: omnidirectional RFs were now found in only 6% (4/68) of the neurons studied (there was no significant difference from the normally-reared incidence: χ^2^ tests, χ^2^ = 0.253, P = 0.615). This caused the average auditory RF size at NE-6 to approximate that found in normal animals (mean diameter = 70.22 ± 26.22°, Fig. 4F). Visual and auditory RF sizes remained stable thereafter.

#### RF register

The auditory RFs of noise-reared animals showed poorer register with their visual counterparts than is typically observed in normally-reared animals (see Fig. 3). To quantify the degree of this RF misalignment, visual and auditory spatial response profiles were first fit with Gaussian functions, normalized, and the lack of register (i.e., misalignment) between these “density” functions calculated as a *t*-score (see Methods). Higher t-scores indicated greater misalignment and thus poorer register. The *t*-scores in noise-reared animals at baseline were significantly higher than in normal animals (noise-reared: 1.38 ± 0.17; normal: 0.58 ± 0.11; Mann-Whitney U tests, P ≤ 0.001) (Fig. 4G). Unlike RF size, RF register did not change significantly after 6 months of normal housing (NE-6) (Mann-Whitney U tests, P = 0.365), and remained anomalous when compared to normal even after an additional 6 months of normal experience (NE-12) (Mann-Whitney U tests, P ≤ 0.001). When, however, animals were given controlled cross-modal exposures during this additional 6 month period, RF register significantly improved (Mann-Whitney U tests, P ≤ 0.001), reaching an average value (0.55 ± 0.05) equivalent to that of normal animals (Mann-Whitney U tests, P = 0.911).

### Multisensory Integration

Consistent with previous reports^6^, noise-rearing had little effect on the incidence of multisensory neurons (53% were multisensory at baseline). There were also no apparent deviations from normal in the robustness with which multisensory neurons responded to visual and auditory stimuli (although this was not examined with a systematic test battery). Nevertheless, the majority (78%, 25/32) of multisensory neurons tested at baseline failed to show multisensory integration capabilities. The typical multisensory response to spatiotemporally concordant visual-auditory stimuli was not enhanced, and the average magnitude of the multisensory enhancement index (ME) (22.75 ± 7.76%) was significantly (Mann-Whitney U tests, P ≤ 0.001) below normal (86.12 ± 7%). This developmental defect was apparent even when the cross-modal stimuli were tested at a variety of locations within their respective RFs.

There was a marginal but non-significant change in the incidence of multisensory neurons exhibiting multisensory integration capabilities after 6 months of normal experience (NE-6), increasing to 35% (24/68; χ^2^ tests, χ^2^ = 1.258, P = 0.262). But, even at this time the incidence remained far below that observed in normal animals (χ^2^ tests, χ^2^ = 27.693, P ≤ 0.001). Defects were also evident in the lack of significant change in the mean ME at NE-6 across the population of neurons studied (baseline = 22.75 ± 7.76% vs. NE-6 = 36.25 ± 5.48%; Mann-Whitney U tests, P = 0.089). At NE-6, the mean ME was still significantly (Mann-Whitney U tests, P ≤ 0.001) below normal. The persistence of the multisensory integration defect despite 6 months of access to the rich variety of cross-modal events in the normal housing condition was striking. This period is far longer than that required for neonatal animals to develop multisensory integration capabilities in such an environment^8^.

**Figure 5:**
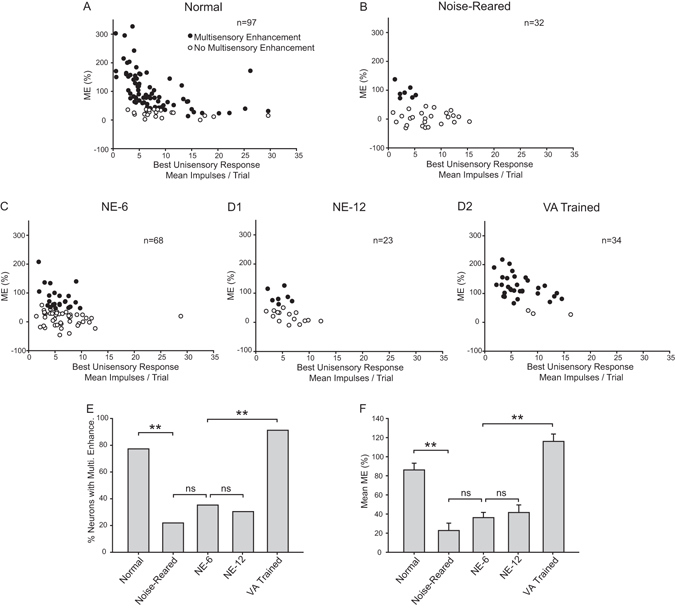
Population comparison of the relative effectiveness of normal housing versus cross-modal exposure in instantiating multisensory integration capabilities. (**A**) A high proportion of neurons exhibit multisensory enhancement capabilities in normal animals. (**B**) However, few SC neurons in noise-reared animals exhibited this capacity. (**C**) This proportion increased somewhat, but not significantly, when those animals were subsequently housed in a normal environment for 6 months (NE-6). (**D1**) This proportion also failed to change significantly in animals remaining in that housing condition for an additional 6 months (NE-12). (**D2**) However, the animals given once weekly cross-modal exposure sessions over an equivalent period did achieve a normal incidence of neurons with this capacity (VA trained). **E** and **F**) Group differences were evident in the summary bar graphs which compare the incidences of multisensory enhancement (**E**), and the mean ME magnitudes (**F**) collapsed over the last 1.5 months of testing. n = number of neurons.

This defect persisted even after up to 6 additional months in the normal housing environment (NE-12). Thus, the summary comparisons in these animals during the last 1.5 months in this environment revealed no significant increase in the incidence of neurons with multisensory integration capabilities (NE-6 = 35% (24/68) vs. NE-12 = 30% (7/23); χ^2^ tests, χ^2^ = 0.0291, P = 0.865) nor in their mean ME (NE-6 = 36.25 ± 5.48% vs. NE-12 = 41.61 ± 7.83%; Mann-Whitney U tests, P = 0.446). In contrast, neurons in the VA Trained group during this same time frame showed a marked increase in the incidence of multisensory integration (NE-6 = 35% (24/68) vs. VA Trained = 91% (31/34); χ^2^ tests, χ^2^ = 13.19, P ≤ 0.001) and potency (ME) (NE-6 = 36.25 ± 5.48% vs. VA Trained = 116.01 ± 7.68%; Mann-Whitney U tests, P ≤ 0.001). Indeed, the incidence of neurons showing multisensory integration in the VA Trained group was not significantly different from that in normally-reared adults (Normal = 77% (75/97) vs. VA Trained = 91% (31/34); χ^2^ tests, χ^2^ = 2.297, P = 0.13) and their MEs were slightly higher (Normal = 86.12 ± 7.00% vs. VA Trained = 116.01 ± 7.68%; Mann-Whitney U tests, P ≤ 0.001) (Fig. 5).

**Figure 6:**
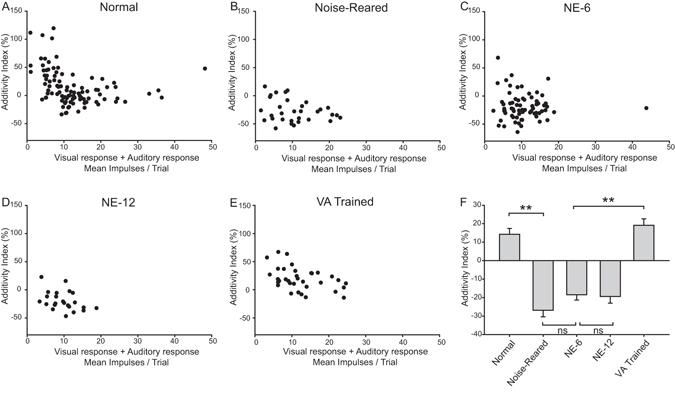
The Additivity Index reveals that the persistence of the multisensory integration deficit induced by noise-rearing is reversed by cross-modal (VA) training. This index uses the sum of the comparator unisensory responses as a benchmark with which to evaluate multisensory integration. Note the difference between the normal scatter plot (**A**) (each point represents a single neuron) and those after noise-rearing (**B**), and after 6 and 12 months of housing in a normal environment (**C**,**D**). Only after cross-modal training does the scatter plot approximate that seen in the normal animal (**E**).

These differences were not influenced by differences in unisensory response magnitude: there were no significant differences in the magnitude of the auditory responses (One Way ANOVA on Ranks, P = 0.575, Normal: 5.81 ± 0.46; Noise: 5.21 ± 0.59; NE-6: 5.16 ± 0.32; NE-12: 4.77 ± 0.40; VA trained: 6.28 ± 0.59) or visual responses (One Way ANOVA on Ranks, P = 0.599, Normal: 6.47 ± 0.58; Noise: 5.36 ± 0.64; NE-6: 5.46 ± 0.46; NE-12: 4.96 ± 0.52; VA-trained: 6.13 ± 0.54) among the five different experimental groups. In addition, consistent results were obtained when using the Additivity Index as a metric, which uses the sum of the visual and auditory unisensory responses as a benchmark against which to evaluate the magnitude of multisensory enhancement (see Fig. 6).

As is typical in normally-reared animals, neurons in the VA Trained group also showed multisensory enhancement to a wide variety of stimuli not presented during the exposure periods. For quantitative comparison, a subgroup (n = 16) was tested with the exposure set (moving bar of light + broadband noise) and a non-exposure set (flashing light spot + pure tone) to examine the generalization of multisensory integration. No significant difference in the mean ME scores between the two stimulus sets was observed (Mann-Whitney U tests, P = 0.536).

### The Timeline of Changes during Cross-Modal Exposure

As noted above, following testing at NE-6, animals were divided into the NE-12 “control” group and the VA Trained group. These groups were maintained and tested in the same way, except the latter was given controlled cross-modal exposures. The groups were not statistically distinguishable on any of the relevant metrics at the time they were formed, but their multisensory development differed significantly over the next 6 months.

**Figure 7:**
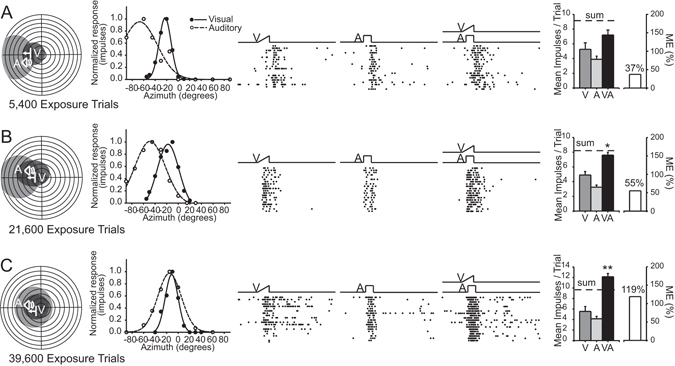
Exemplars illustrate a gradual development of multisensory integration capabilities, and receptive field register, during the cross-modal exposure period. Shown are 3 typical neurons recorded from the same noise-reared animal after being exposed to an increasing number of cross-modal stimuli. Below each schematic are the numbers of exposures (5,400 trials ≈ 9 hours of experience; 21,600 ≈ 36 hours; 39,600 ≈ 66 hours) provided prior to the tests generating these data. Conventions are the same as in Figs 2 and 3. Left: The RFs and visual and auditory spatial response profiles increased their overlap with increased cross-modal exposure. Middle: rasters and summary graphs show progressive increases in multisensory enhancement with increasing cross-modal exposure. Right: Summary of the changes in responsiveness and ME. *P < 0.05. Conventions are the same as in previous figures.

The typical progression of RF register and multisensory integration with training is illustrated by three exemplar neurons in Fig. 7. The neuron with the fewest exposure trials (3 exposure sessions; 5,400 exposures) had misaligned cross-modal RFs, and its multisensory response magnitude failed to significantly exceed its response to the most effective modality-specific component stimulus (Fig. 7A). In contrast, neurons with greater numbers of training sessions showed progressively greater RF register and multisensory enhancement magnitudes. This is evident in the neurons tested after 12 training sessions (21,600 trials, Fig. 7B) and 22 sessions (39,600 trials, Fig. 7C). In the latter neuron the response now significantly exceeded the sum of the two unisensory comparator responses, which is typical of normal multisensory integration when weakly effective cross-modal stimuli are presented^9–12^.

**Figure 8:**
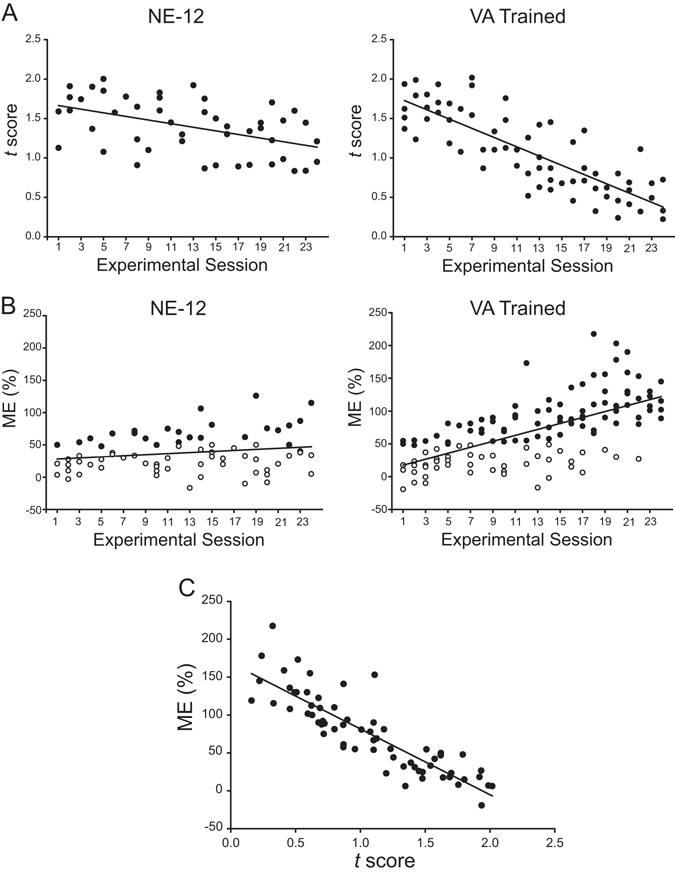
Population data showing the time course of receptive field register and multisensory integration capabilities over the cross-modal exposure sessions relative to controls. (**A**) While the *t*-score representing visual-auditory RF register remained high (misalignment) in noise-reared animals subsequently housed in the normal environment for 12 months (NE-12), the *t* score in animals in the cross-modal trained group gradually decreased. (**B**) Similarly, while there was no significant increase in either the incidence of neurons showing multisensory integration capabilities (filled circles) or their enhancement index in the NE-12 animals, these scores gradually increased to reach adult-like levels for neurons in the VA trained group. (**C**) Improvements in RF register and ME were highly correlated on a sample-by-sample basis: neurons with greater levels of RF register more likely to show higher levels of multisensory enhancement (r = −0.8).

The timeline of these changes in RF register and the magnitude of multisensory enhancement with cross-modal exposure were reflective of the population trends as shown in Fig. 8. After 13 training sessions, the regression line in the cross-modal exposure group entered the 95% confidence intervals of the distribution of ME from the normal cohort. Extrapolating from the fitted regression line, the NE-12 control group would not have entered these intervals until after 40 weeks.

The parallel trends in the development of RF register and multisensory integration with cross-modal exposure (Fig. 8A and B) suggest the presence of a common mechanism driving their maturation. This is underscored by the significant negative correlation between the *t*-score and ME (r = −0.87, P < 0.0001) in these neurons (Fig. 8C).

## Discussion

The present study demonstrated that the development of visual-auditory integration capabilities, when precluded by noise-rearing, was initiated in adulthood by giving animals repeated exposure to paired visual-auditory cues in a laboratory setting. These observations are consistent with those from studies in which this multisensory maturational process was initiated by cross-modal training after having been precluded by dark-rearing^5^. Although the two approaches to compromising cross-modal experience and the development of multisensory integration capabilities were quite different, the multisensory defects they induced were the same, and were resolved with equal rapidity by the same cross-modal training paradigm. In both cohorts, the resolution of the multisensory defect with training followed nearly identical linear trends, reaching normal adult-like performance levels after approximately the same amount of cross-modal exposure. This suggests that the specific manner by which the defect is introduced does not differentially impact the potential for the later acquisition of this capacity, and its development can proceed with high efficiency even in adulthood.

It is unlikely that training with visual or auditory cues alone would have been effective at driving the development of these enhancement capabilities. This development is neither spurred by training dark-reared animals with modality-specific visual and auditory stimuli^5^, nor by exposing neonates to random visual and auditory stimuli^13^.

Also evident from these datasets was that neither RF contraction nor RF register, each of which normally correlates with the development of multisensory integration^8, 14^ has a causative relationship with it. The conclusion regarding RF contraction is based on the observation that animals in the dark-rearing study were able to develop their multisensory integration capabilities when given cross-modal training despite maintaining enlarged RFs. Apparently, the visual and auditory experience provided by these exposure sessions (they were housed in the dark at all other times) was insufficient for this unisensory property to mature. In contrast, in the present noise-rearing studies, animals that were moved to standard housing for 6 months showed RF contraction despite a failure to develop multisensory integration capabilities. Thus, RF contraction appears to be linked to unisensory experience, but not multisensory experience^6^. In contrast, both multisensory integration and the spatial register of a neuron’s visual and auditory RFs require extensive multisensory experience^1^. Nevertheless, multisensory integration capabilities can develop regardless of whether a neuron’s cross-modal RF register becomes good or poor^15^.

Despite the lack of a causal relationship between these two RF properties and multisensory integration, it is noteworthy that under normal conditions they do develop in tandem. As such, under normal developmental circumstance RF properties can be very good prognostic tools for assessing the likelihood that a given neuron had developed its capacity to integrate its cross-modal inputs.

It is interesting to note that although there were no statistically significant changes in the incidence or potency of multisensory enhancement in the groups that were placed in the normal environment for 6 or 12 months, there was a slight, albeit marginally non-significant, positive trend in both metrics. Based on the slope and intercept of the regression line across those groups, it was predicted that the normal levels of multisensory potency and incidence would be reached after nearly 1.5 years in that environment. Interestingly a “spontaneous remission” of multisensory processing defects has also been noted in high-functioning ASD individuals by Foxe and colleagues^16^, but also requires years to do so.

The contrast between the effectiveness of the exposure paradigm and the normal environment found here also helps resolve a puzzle arising from prior studies. Whereas Rowland *et al*. found that multisensory defects induced by cortical deactivation early in life took 1.5–4 years to resolve in a normal environment^2^, Yu *et al*. were able to resolve it in dark-reared animals after only a few hours of multiple visual-auditory exposure sessions in the laboratory^5^. The present results suggest that these different time scales did not reflect differences in how this developmental process was disrupted, but rather the circumstances in which later cross-modal experience was obtained, and the statistical properties of that experience^1, 17^. While stimuli in normal environments are quite variable in their features and relationships, the cross-modal stimuli experienced in the visual-auditory exposure paradigm were always the same, they always appeared and ended at the same time, and were always in the same locations in space. This high degree of covariance was likely a significant factor in instantiating multisensory integration capabilities following sensory restriction.

Given that the impressive neonatal unisensory plasticity normally degrades later in life^18–21^, it is likely that a similar age-related decline in multisensory plasticity would have been incurred by the animals reared without visual-auditory cross-modal experience. This might explain the apparent maturational shift in the cross-modal experiential requirements for their development of multisensory integration capabilities. Whereas the neonate may be capable of rapidly incorporating perceived covariation in cross-modal signals even when that covariation is not precise, the naïve adult may require a much higher level of consistency and covariation (even if only present within the few hours of the exposure paradigm) to initiate the same developmental process. If so, then the very richness of normal environments, which typically facilitates the maturation of sensory systems^22–25^, would become an impediment to the adult development of multisensory integration.

The developmental inertia in these adults naïve to visual-auditory events might also reflect an “alternative” developmental trajectory that has to be overcome. For example, early visual restriction has been shown to lead to opportunistic expansion of auditory afferents into what would otherwise be visual areas of association cortex^26, 27^, and early auditory restriction facilitates the invasion of visual afferents into primarily auditory areas^28^. These and other effects of sensory restriction are likely to have crafted a circuitry within AES and an AES-SC synaptic architecture that would take time to reorganize for it to begin facilitating multisensory integration.

Underscoring this issue is the likelihood that many sensory events in the normal environment would encourage the retention of whatever opportunistic circuits had already been formed. For example, if the absence of correlated visual-auditory experience embeds a principle in the circuit that visual and auditory “events” are always independently-sourced, every independently-sourced visual or auditory event in the normal environment, of which there are surely many, would reinforce the established plan. This would create resistance to its alteration. Having to overcoming this resistance would further slow the acquisition of multisensory integration capabilities despite its being guided by the same basic principle: the statistical properties of cross-modal experience^13^.

These observations also suggest that the normal developmental trajectory with which multisensory integration capabilities are acquired would be accelerated by a cross-modal training program during early life. But this remains to be demonstrated. In addition, the results have clear implications for the rehabilitative strategies used in human patients recovering from early visual and/or auditory deficits. They suggest that engaging a systematic cross-modal training program will have better multisensory outcomes than relying on the inherent richness of a normal sensory environment^29–31^. Such active cross-modal training strategies may also induce related changes in widespread areas of the nervous system where multisensory integration normally takes place^31–34^ and which may have also been compromised by the rearing condition.

## Methods

### Animal groups

Protocols were in accordance with the NIH Guide for the Care and Use of Laboratory Animals Eighth Edition (NRC, 2011), and were approved by the Animal Care and Use Committee of Wake Forest School of Medicine, an Association for the Assessment and Accreditation of Laboratory Animal Care International-accredited institution.

Five cats (3 males and 2 females from 2 litters) were deprived of normal auditory experience (and thus auditory-visual experience) by rearing them from birth to 6 months of age in an omnidirectional noise room. Continuous omnidirectional broadband noise (80 dB) was provided from speakers located on all sides and above their pen^6^. When the animals were 6 months of age (at which time multisensory processing in the neurotypic SC is adult-like), they were implanted with a recording well to provide access to the SC without wounds or pressure points^35^. Initial weekly recording sessions to establish a “baseline” electrophysiological dataset began after at least 7 days of post-surgical recovery. In each case, animals were anesthetized in their housing environment and transported to the laboratory in a covered carrier to minimize their exposure to auditory-visual cues. They were returned to their housing environment via the same transport carrier immediately after each recording session. After approximately 3 weeks of these baseline noise-reared tests, they were moved to the standard animal housing facility. This environment was replete with a rich variety of visual-auditory cues and was, for all operational purposes, equivalent to that of other laboratory animals used in studies of the features and maturation of SC multisensory integration^1^. It is referred to hereafter as the “normal” environment.

After 6 months of housing in this environment, they were tested again to assay the effectiveness of normal experience in facilitating the development of SC multisensory integration capabilities after noise-rearing (Fig. 1). This established a second baseline, in this case after 6 months of noise-rearing plus 6 months of subsequent normal experience (referred to hereafter as “NE-6”).

After these tests, animals remained in the normal environment, but were divided into two groups for further experimentation. In this final phase, they were tested weekly for an additional 6 months (Fig. 1). The first group of animals, the training group (“VA Trained,” n = 3) received repeated exposure to spatiotemporally coincident visual-auditory cross-modal stimuli in the electrophysiology suite prior to each weekly recording session. The second group (“NE-12,” n = 2) was treated identically except the animals did not receive the cross-modal exposures prior to each weekly recording sessions. They simply had a second 6 month period of normal housing.

This final stage of experimental testing was used to compare whether the addition of cross-modal exposure sessions facilitated the development of SC multisensory integration over the same time period. Although the development (or lack of development) was tracked throughout this final 6 month period, for the purposes of comparing the populations of neurons in the VA Trained and NE-12 groups, only data collected during the last 1.5 months (i.e., after 4.5 months of exposure or normal housing) were used in the summary analyses. Thus, although the “control” group is labeled NE-12, operationally the summary data were acquired in a window between 10.5 and 12 months of experience in the normal environment.

### Cross-modal exposure sessions

Each animal in the VA Trained group was repeatedly exposed to spatiotemporally concordant visual and auditory (cross-modal) stimuli (1800 exposures/week; 6 sec inter-stimulus interval) in the electrophysiology suite prior to each weekly recording session. The locations of these cross-modal stimuli were randomly varied between 5 selected locations in space, for a total of 360 exposures/location/weekly 3 hour session (see Fig. 2). The visual component stimulus was a moving bar of light (10° × 2°, 13.5 cd/m^2^ against a 0.86 cd/m^2^ background, speed = 100°/s, duration = 100 ms). The auditory component stimulus was a brief (100 ms) burst of broadband noise (20–20,000 Hz, 65 dB) against an ambient background of 51.4~52.7 dB. The physiological recording session began at the end of the exposure session. All neurons sampled had both visual and auditory RFs overlapping at least one exposure site and in approximately 20% they overlapped >1 site.

### Surgery and electrophysiological testing procedures

#### Surgery

Animals were anesthetized with ketamine hydrochloride (20 mg/kg, i.m.) and acepromazine maleate (0.1 mg/kg, i.m.), intubated, and placed in a stereotaxic apparatus in the surgical suite. Surgical anesthesia was then induced with isoflurane (2–4%) and maintained with isoflurane (1.5–2%). During surgery, expiratory CO_2_, SpO_2_, blood pressure, and heart rate were continuously monitored using a digital vital signs monitor (VetSpecs VSM7), and body temperature was maintained at 37–38 °C with a heating pad. A stainless steel recording chamber was placed over a craniotomy to provide access to the SC via the overlying cortex and attached to the skull with stainless steel screws and dental acrylic^35^. Postsurgical analgesics (ketoprofen, 2 mg/kg, i.m.) were administered as needed, and antibiotics (ceftriaxone, 20 mg/kg, i.m.) were administered twice a day for 7 days.

#### Electrophysiological testing procedure

Electrophysiological testing procedures were the same as those used previously^5, 6, 13^. For each session, the animal was anesthetized with ketamine hydrochloride (20 mg/kg, i.m.) and acepromazine maleate (0.1 mg/kg, i.m.), intubated, and ventilated mechanically. It rested in a comfortable recumbent position and its head orientation was fixed by two removable horizontal stainless steel bars that attached the implanted recording chamber to a metal frame without the introduction of wounds or pressure points. Respiratory rate and volume were adjusted to keep the end-tidal CO_2_ at ~4%. Expiratory CO_2_, SpO_2_, heart rate, and blood pressure were monitored continuously to ensure the maintenance of anesthesia. Paralysis was induced with an infusion of pancuronium bromide (0.1 mg/kg, i.v.) to prevent ocular drift. Anesthesia, paralysis, and hydration were maintained by continuous intravenous infusion of ketamine hydrochloride (5–10 mg kg^−1^h^−1^), pancuronium bromide (0.05 mg kg^−1^h^−1^), and 5% dextrose in sterile saline (2.4–3.6 ml/h). Body temperature was kept at 37–38 °C via a circulating hot water heater.

Glass-coated tungsten electrodes (1–3 MΩ at 1 kHz) were used to record single neurons in the multisensory (i.e. deep) layers of the SC. Sensory responsive neurons were identified using a variety of visual and auditory search stimuli. Visual search stimuli consisted of moving bars of light that were back-projected from an LC4445 Philips onto a tangent screen placed in front of the animal. Auditory search stimuli consisted of broadband (20–20,000 Hz) noise bursts from any of 16 hoop-mounted speakers placed 15° apart and 15 cm from the animal’s head. Once a visual-auditory neuron was identified, its receptive fields (RFs) were mapped using standard methods^6^. The unisensory and multisensory responses of these neurons were then examined and quantified using randomly-interleaved presentations of modality-specific and spatiotemporally concordant cross-modal stimuli at inter-trial intervals of 5–7 s, with 20 trials per condition. The stimulus parameters used to study neurons in each group were equivalent and the same as those used previously^6^. Visual stimuli (100–200 ms duration) were rectangular bars of light (6° × 2°) of varying intensity (1.1–13.5 cd/m^2^ against a 0.86 cd/m^2^ background) and moved in the optimal direction and speed for each neuron as determined during a series of visual pre-tests. The auditory stimuli consisted of brief (100–200 ms) broadband noise bursts (20–20,000 Hz) of varying intensity (55–70 dB, against an ambient background of 51.4~52.7 dB). A brief series of preliminary evaluations in which stimulus intensities were systematically manipulated were used to choose stimulus intensity levels that were weakly effective, maximizing the likelihood of exposing the multisensory integration capability of the particular neuron under study^9^. These tests also identified the auditory response threshold, defined as the auditory intensity that evoked a significant response on only 50% of the stimulus presentations. In some neurons, combinations involving a greater variety of visual and auditory cues (e.g., flashes, tones) were also tested to determine whether observed integration capabilities were specific to particular stimulus features^13^.

When time permitted, the modality-specific visual and auditory spatial tuning within a neuron’s RFs was examined by randomly-varying the location of an effective modality-specific stimulus in azimuth and recording the neuron’s response magnitude^6^. For examination of visual spatial response profiles, a rectangular moving bar (4° × 1°) was used. Its intensity was 16.5 cd/m^2^ presented against the uniform dark background and moved at 60°/s through a tightly restricted region (3°). For examination of auditory spatial response profiles, a broadband noise burst 10 dB above the threshold was used. The mean number of impulses evoked by 20 repetitions of each stimulus was used to construct a spatial response profile for each modality.

### Analysis

Impulse times (1 ms resolution) were recorded for each trial and analyzed off-line. The response window was defined using the algorithm identified in earlier studies^36^. The magnitude of each response was identified as the mean number of impulses occurring in the response window minus the expected number given the spontaneous firing rate (mean spontaneous firing rate for each condition was calculated in the 500 ms window preceding the stimulus). The response to the combined cross-modal stimuli was statistically compared with the response evoked by the more effective individual component (i.e., modality-specific) stimulus to determine if the neuron’s response was significantly enhanced (*t* test, alpha = 0.05). Multisensory enhancement was used as the index of multisensory integration, as it has been shown to be the most sensitive index of an SC neuron’s multisensory integration capabilities^37^. It was operationally defined as a significant increase in the mean number of impulses elicited by the cross-modal stimulus compared with those elicited by its most effective modality-specific component stimulus. The magnitude of multisensory enhancement (ME) was evaluated by the formula: ME = [(CM − SM_max_)/SM_max_] × 100, where CM represents the mean magnitude of the multisensory response, and SM_max_ represents the magnitude of the response evoked by the more effective modality-specific stimulus^38^. The magnitude of multisensory enhancement was also evaluated by the Additivity Index. Additivity Index = [(CM − SM_vis_ − SM_aud_)/(SM_vis_ + SM_aud_)] × 100. SM_vis_ represents mean magnitude of visual response, and SM_aud_ represents mean magnitude of auditory response. Incidence values were compared across groups using χ^2^ tests, ME values were compared using Mann-Whitney U tests.

Spatial response profiles were normalized by the maximum response and fit with Gaussian functions. The degree of register of the visual and auditory spatial response profiles was then quantified as a *t*-score:

$$t=\frac{|{X}_{a}-{X}_{v}|}{\sqrt{{\sigma }_{a}^{2}+{\sigma }_{v}^{2}}}$$

where *X*_*a*_ and *X*_*v*_ are the locations of the peaks of the Gaussian fits to the auditory spatial and visual spatial response profiles, and *σ*_*a*_ and *σ*_*v*_ are the standard deviations. The lower the *t*-score, the higher the degree of visual-auditory spatial register.

The diameters of spatial response profiles are reported as mean scores with standard deviations (±), average response magnitudes and ME are reported as mean scores with standard errors (±). Neuronal incidences were compared using χ^2^ tests, response magnitudes were compared using *t*-tests, relationships were evaluated using regression, and spatial response profile diameters, RF register (i.e., *t*-scores), ME scores, and Additivity Index were compared between cohorts using Mann-Whitney U-tests.

## References

[1] Stein BE, Stanford TR, Rowland BA (2014). Development of multisensory integration from the perspective of the individual neuron. Nat Rev Neurosci.

[2] Rowland BA, Jiang W, Stein BE (2014). Brief cortical deactivation early in life has long-lasting effects on multisensory behavior. J Neurosci.

[3] Jiang W, Jiang H, Rowland BA, Stein BE (2007). Multisensory orientation behavior is disrupted by neonatal cortical ablation. J Neurophysiol.

[4] Wallace MT, Perrault TJ, Hairston WD, Stein BE (2004). Visual experience is necessary for the development of multisensory integration. J Neurosci.

[5] Yu L, Rowland BA, Stein BE (2010). Initiating the development of multisensory integration by manipulating sensory experience. J Neurosci.

[6] Xu J, Yu L, Rowland BA, Stanford TR, Stein BE (2014). Noise-rearing disrupts the maturation of multisensory integration. Eur J Neurosci.

[7] Xu J, Yu L, Stanford TR, Rowland BA, Stein BE (2015). What does a neuron learn from multisensory experience?. J Neurophysiol.

[8] Wallace MT, Stein BE (1997). Development of multisensory neurons and multisensory integration in cat superior colliculus. J Neurosci.

[9] Meredith MA, Stein BE (1986). Visual, auditory, and somatosensory convergence on cells in superior colliculus results in multisensory integration. J Neurophysiol.

[10] Wallace MT, Meredith MA, Stein BE (1998). Multisensory integration in the superior colliculus of the alert cat. J Neurophysiol.

[11] Perrault TJ, Vaughan JW, Stein BE, Wallace MT (2005). Superior colliculus neurons use distinct operational modes in the integration of multisensory stimuli. J Neurophysiol.

[12] Stanford TR, Quessy S, Stein BE (2005). Evaluating the operations underlying multisensory integration in the cat superior colliculus. J Neurosci.

[13] Xu J, Yu L, Rowland BA, Stanford TR, Stein BE (2012). Incorporating cross-modal statistics in the development and maintenance of multisensory integration. J Neurosci.

[14] Jiang W, Jiang H, Stein BE (2006). Neonatal cortical ablation disrupts multisensory development in superior colliculus. J Neurophysiol.

[15] Wallace MT, Stein BE (2007). Early experience determines how the senses will interact. J Neurophysiol.

[16] Foxe JJ (2015). Severe multisensory speech integration deficits in high-functioning school-aged children with Autism Spectrum Disorder (ASD) and their resolution during early adolescence. Cereb Cortex.

[17] Beierholm UR, Quartz SR, Shams L (2009). Bayesian priors are encoded independently from likelihoods in human multisensory perception. J Vis.

[18] Hubel DH, Wiesel TN (1962). Receptive fields, binocular interaction and functional architecture in the cat’s visual cortex. J Physiol.

[19] Edeline JM, Pham P, Weinberger NM (1993). Rapid development of learning-induced receptive field plasticity in the auditory cortex. Behav Neurosci.

[20] Bao S, Chan VT, Merzenich MM (2001). Cortical remodelling induced by activity of ventral tegmental dopamine neurons. Nature.

[21] Zhang LI, Bao S, Merzenich MM (2001). Persistent and specific influences of early acoustic environments on primary auditory cortex. Nat Neurosci.

[22] Sale A, Berardi N, Maffei L (2009). Enrich the environment to empower the brain. Trends Neurosci.

[23] Bengoetxea H (2012). Enriched and deprived sensory experience induces structural changes and rewires connectivity during the postnatal development of the brain. Neural Plast.

[24] Maya-Vetencourt JF, Origlia N (2012). Visual cortex plasticity: a complex interplay of genetic and environmental influences. Neural Plast.

[25] Chaudhury S, Sharma V, Kumar V, Nag TC, Wadhwa S (2016). Activity-dependent synaptic plasticity modulates the critical phase of brain development. Brain Dev.

[26] Rauschecker JP (1995). Compensatory plasticity and sensory substitution in the cerebral cortex. Trends Neurosci.

[27] Collignon O (2015). Long-Lasting Crossmodal Cortical Reorganization Triggered by Brief Postnatal Visual Deprivation. Curr Biol.

[28] Lomber SG, Meredith MA, Kral A (2010). Cross-modal plasticity in specific auditory cortices underlies visual compensations in the deaf. Nat Neurosci.

[29] Bolognini N, Rasi F, Coccia M, Ladavas E (2005). Visual search improvement in hemianopic patients after audio-visual stimulation. Brain.

[30] Putzar L, Goerendt I, Lange K, Rosler F, Roder B (2007). Early visual deprivation impairs multisensory interactions in humans. Nat Neurosci.

[31] Jiang H, Stein BE, McHaffie JG (2015). Multisensory training reverses midbrain lesion-induced changes and ameliorates haemianopia. Nat Commun.

[32] Wallace MT, Ramachandran R, Stein BE (2004). A revised view of sensory cortical parcellation. Proc Natl Acad Sci USA.

[33] Ghazanfar AA, Schroeder CE (2006). Is neocortex essentially multisensory?. Trends Cogn Sci.

[34] Dundon NM, Bertini C, Ladavas E, Sabel BA, Gall C (2015). Visual rehabilitation: visual scanning, multisensory stimulation and vision restoration trainings. Front Behav Neurosci.

[35] McHaffie JG, Stein BE (1983). A chronic headholder minimizing facial obstructions. Brain Res Bull.

[36] Rowland BA, Stein BE (2007). Multisensory integration produces an initial response enhancement. Front Integr Neurosci.

[37] Kadunce DC, Vaughan JW, Wallace MT, Benedek G, Stein BE (1997). Mechanisms of within- and cross-modality suppression in the superior colliculus. J Neurophysiol.

[38] Meredith MA, Stein BE (1983). Interactions among converging sensory inputs in the superior colliculus. Science.

